# The Effect of Age on Thymic Function

**DOI:** 10.3389/fimmu.2013.00316

**Published:** 2013-10-07

**Authors:** Donald B. Palmer

**Affiliations:** ^1^Infection and Immunity Group, Department of Comparative Biomedical Sciences, Royal Veterinary College, University of London, London, UK

**Keywords:** thymus, immunosenescence, thymic involution, thymic stroma, thymocyte

## Abstract

Age-related regression of the thymus is associated with a decline in naïve T cell output. This is thought to contribute to the reduction in T cell diversity seen in older individuals and linked with increased susceptibility to infection, autoimmune disease, and cancer. Thymic involution is one of the most dramatic and ubiquitous changes seen in the aging immune system, but the mechanisms which underlying this process are poorly understood. However, a picture is emerging, implicating the involvement of both extrinsic and intrinsic factors. In this review we assess the role of the thymic microenvironment as a potential target that regulates thymic involution, question whether thymocyte development in the aged thymus is functionally impaired, and explore the kinetics of thymic involution.

## The Impact of Thymic Involution on Peripheral T Cell Senescence

Advance aging correlates with a reduced ability of the immune system to generate antigen specific responses to pathogens and vaccination. This collectively results in a higher incidence of infection, neoplastic, and autoimmune diseases which are preferentially observed in older individuals. These profound changes exhibited by the aging immune system is termed immunosenescence, which affects both innate and adaptive immunity (1–3).

The thymus is responsible for the development of self-restricted, self-tolerant, immunocompetent T cells but has no self-renewal properties relying on the continuous replenishment of new T cell progenitors from the bone marrow. Maturation of these cells occur through a series of proliferation and differentiation stages dependent upon receiving instructions from the specialized thymic microenvironment (4, 5).

**Figure 1:**
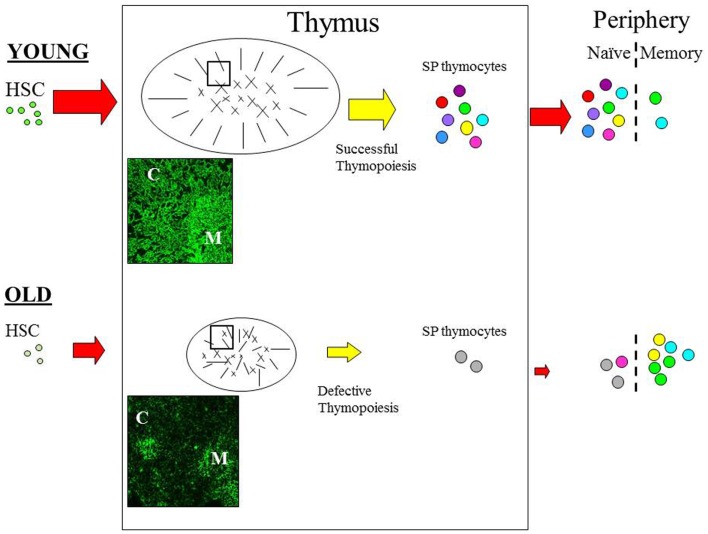
**The effect of age on thymic function**. Schematic diagram outlining the pathway of T cell development. Aging can impact on variety of pathways during the development of T cells. With increasing age HSC appear to have a reduced lymphoid potential and increased myeloid differential capacity. Age-related involution is associated with reduced thymic mass and altered architecture resulting in reduced thymic output in the aged thymus. In the young, T cell development is functional and the peripheral T cell is pool is diverse; as depicted by the various colors. Furthermore, normal thymopoiesis provides a positive effect on thymic structure; thymic cross-talk. In contrast, T cell output is significantly reduced in the aged thymus resulting in loss of diversity and alteration in the phenotype and function of peripheral T cells; with the majority of cells being in the memory pool. Modifications in the thymic microenvironment are likely to have an impact on thymopoiesis resulting in defective RTE, which in turn disrupt thymic cross-talk leading to further alteration of TEC structure. Immunofluorescence image of young (6 weeks) and old (18 months) murine thymus stained with anti-keratin antibody (55) which detects cortical (C) and medullary (M) TEC. In the young thymus, antibody staining shows the cortical epithelium as a network of long thin processes, while the medullary region reveals a squamous appearance. In contrast, the staining on old thymic section revealed a reduced network of cortical epithelial cells, the medullary region is smaller and more diffuse while the cortical-medullary junction is less distinctive; as also depicted in the schematic. C, cortex; M, medulla. Picture (100× magnification).

One of the most acknowledged changes of the aging immune system is regression, or involution of the thymus (6–8), which seems to occur in almost all vertebrates suggesting that this is an evolutionary ancient and conserved process (9). Age-associated thymic involution involves a decrease in tissue mass and cellularity, together with a loss of tissue organization with the net outcome being a reduction in naïve T cell output [Figure 1; (6–8)]. This decline in naïve T cell output is believed to have a major impact on the properties on the peripheral T cell pool such that with increasing age, these cells exhibit alterations in phenotype and function, loss of diversity, and replicative senescence (10, 11). Moreover, it is these age-related changes in peripheral T cells that are believed to contribute significantly toward the features of immunosenescence (12, 13), suggesting that the altered thymic activity is a key trigger toward the decline of immune function in the aged (14).

While animal models show that the maintenance of naïve peripheral T cells in the adult do indeed require the release of cells from the thymus (15, 16). In humans, however the relationship between thymic activity and naïve T cell homeostasis is a matter of debate, with the recent observations that peripheral proliferation and not thymic output contributes to the maintenance of naïve T cells in young adults (17). Nevertheless, using signal-joint T cell receptor (TCR) excision circles (sjTREC) as a measurement of thymic function, numerous studies have shown lower sjTREC levels in elderly individuals are associated with a reduction of naïve T cells (18–20).

Moreover, a direct correlation between thymic function and naïve T cell number comes from studies examining the peripheral immune system of thymectomized individuals (21). In one such study which looked at patients 20+ years after thymectomy, the authors observed a decreased proportion of naïve T cells, reduction in TCR diversity and noted that such changes were more marked in individuals infected with *Cytomegalovirus* (22). Furthermore, thymectomized individuals exhibited a delayed primary response to tick-borne encephalitis vaccination (23). Interestingly, these and other studies seem to suggest that the thymus may play a role in maintaining immune efficacy in the adult (21). Indeed, reports, using mice, have demonstrated the need for the continual production of naïve T cells to mount an effective immune response against bacterial (24), viral (25), and fungal infections (26); with the latter study showing that mice thymectomized at 5 weeks of age exhibited a delayed response to Pneumocystis infection. Furthermore, amongst HIV-infected patients under highly active antiretroviral therapy, those individuals that show enhanced T cell output appear to demonstrate a better prognosis (27, 28). Furthermore, a recent study proposed that thymic function is a key marker in determining mortality in elderly humans (29). Thus, the notion that thymus activity may play an important role in host defense of the adult is interesting and clearly merits further investigation.

## Changes in Thymocyte Development with Age

Although the exact mechanisms involved in age-associated thymic involution are not fully understood, a picture is emerging suggesting defects are present within both developing thymocytes and thymic stroma (30). Thymopoiesis involves a series of sequential developmental steps. Briefly, bone marrow progenitors enter into the thymus and are identified by a lack of both CD4 and CD8. Referred to as double negative (DN) thymocytes, these cells differentiate to become double positive (DP), expressing both CD4 and CD8, and subsequently mature into either single positive (SP) CD4 or SP CD8 T cells, through the process of positive and negative selection, and then exit into the periphery (4, 5).

Given that the thymus requires the continual input of bone marrow progenitors, any age-related alterations in hematopoietic stem cells (HSC) function could conceivably contribute toward thymic involution. Studies have demonstrated that aged HSC appear to exhibit an increased bias toward myeloid differentiation together with a reduced capacity toward lymphoid maturation; which has been observed in mice and human (31, 32). Such alterations in HSC function may manifest within early thymocyte progenitor (ETP) activity. Indeed, aged mice have fewer numbers of ETP, which exhibit reduced proliferation and differentiation potential (33, 34). ETP obtained from young mice are able to differentiate into all the stages of T cell development when seeded into fetal thymic organ culture, in contrast aged ETP showed a reduction of T cell differentiation activity (33). Furthermore, ETP from aged mice show an increased frequency of cells undergoing apoptosis together with a reduced number of Ki67^+^ cells (34). ETP are contained within the earliest stages of DN thymocytes and other studies have highlighted further age-related changes within the later stages of DN thymocyte development; with the observation of a decrease in proportion of CD44^+^CD25^+^ (DN2) and CD44^−^CD25^+^ (DN3) cells (35–38). Additionally, a population of CD44^+^CD24^−^CD3^+^ DN cells has been shown to accumulate in the thymus of older mice (35, 39–41). Interestingly, a similar population has been identified in adult murine bone marrow which appears to be associated with a role in reducing hematopoiesis (42), giving rise to the possibility that the accumulation of such cells in the aging thymus might have a negative impact on thymopoiesis thereby contributing to thymic involution.

Further stages in thymocyte maturation also exhibit phenotypical alterations with age; in particular, studies have demonstrated an age-associated decline of CD3 expression on DP and SP thymocytes (40, 41, 43). Such changes may result in impaired TCR-dependent stimulation. Indeed, it has been demonstrated that aged thymocytes, in comparison to young cells, showed reduced Concanavalin A-induced proliferation (37, 40, 41, 44), with the observation that aged cells failed to enter into the G_2_M phase of the cell cycle (41).

Arguably, these age-related changes in thymopoiesis are likely be acquired by RTE; leading to the possibility that such cells will exhibit reduced immunocompetence. Indeed, several studies have showed that aged RTE undergo phenotypic maturation with delayed kinetics, exhibit decreased proliferative capacity, defective calcium signaling following TCR stimulation, and reduced helper and memory activity (45–47). Furthermore, peripheral T cells from older mice exhibit increased resistance to apoptosis which again may be acquired during thymocyte development as it has been demonstrated that thymocytes from older animals are more resistant to apoptosis (41, 44, 48). It is unlikely that the impairment of aged RTE is acquired in the periphery, but is imprinted during their development in the aged thymus and propose that such flawed cells are also likely to contribute further to peripheral immunosenescence. Moreover, these studies also question, the notion regarding whether T cell development is functionally active in the aged and in light of these studies, this often held view may need to be revised (40).

## Age-Associated Changes in the Thymic Stromal Environment

The thymic stroma plays a crucial role in thymopoiesis by providing the signals necessary to promote proliferation and differentiation due primarily to the influence of cortical and medullary epithelial cells (4); thus age-related changes in the thymic niches could potentially promote thymic involution. In fact, we have argued that the extrinsic defects within the aged microenvironment contribute significantly to age-associated thymic involution (1, 14, 49). Several studies have demonstrated that with age, the thymic microenvironment undergoes structural, phenotypical, and architectural changes (50). This include down regulation of various thymic epithelial cell (TEC) markers such keratin, MHC class II together with alterations of cortical and medullary markers (37, 51–55). Furthermore, the structural integrity of the thymic niche is disrupted with age, including disorganization of the cortical and medullary junction; together with increase fibrosis, adipose tissue, and the accumulation of senescent cells in the aged thymus (40, 55–57).

The age-associated changes in thymopoiesis would principally imply intrinsic defects, however, closer examination reveal that perhaps such alterations could be due, in part, to extrinsic defects within the aged thymic stromal niche resulting in impaired T cell development. For instance, studies have revealed that the production of IL-7, which is necessary for thymopoiesis (58), decreases with age (59). This may be due to the observed loss of MHC class II^+^ TEC in the aged thymus which has been identified as the cell type responsible for producing IL-7 (54). Moreover, IL-7 administered in older mice (60) and rhesus macaques (61) was shown to increased thymic output. Interestingly, bone marrow from young mice injected into lethally irradiated older mice failed to restore thymic architecture and was still accompanied by a reduction in quantitative thymic function (62). In an elegant study addressing the repopulation potential of thymic progenitors, Zhu and colleagues transplanted fetal thymic lobes under the kidney capsule of 1-month-old and 18-months-old mice and observed that the total number and proportion of developing thymocytes in the grafts were similar in older and younger host mice (56, 63). Similar results were obtained when transplanting RAG deficient thymic lobes in that the ability of wild-type thymic progenitors to develop stromal patterning was not dependent on the age of recipients (63). In contrast, it was observed that intrathymic injection of young ETP fail to develop in older animals but did so in the thymus of young recipients (63). Furthermore, recent studies revealed that age-associated thymic involution results primarily with changes in gene expression profile in thymic stromal cells (64).

Above all, these studies suggest that the thymic stroma is a key factor in regulating thymic involution and perhaps the acquired intrinsic defects in aged thymocytes could be due to the inability of the aged thymic microenvironment to support and maintain thymopoiesis (56). Furthermore, the inter-dependency of both thymocyte and TEC to maintain a functional thymic structure (i.e., thymic cross-talk), is also likely to be a contributing factor toward thymic involution (65). Indeed, disrupting the integrity of TEC in the adult thymus has been shown to mimic thymic involution. The transcription factor Foxn1, which is essential for TEC development (66), has been shown to be important for maintaining TEC activity and reducing *Foxn1* expression in the postnatal thymus mimics features of thymic involution (67, 68). In contrast, over expression of Foxn1 delays age-associated thymic involution (69). Moreover, rejuvenation of the aging thymus has been successful when targeting TEC, with the administration of exogenous keratin growth factor being shown to enhance thymic cellularity, restore thymic architecture, and improve immune function in aged mice (70). Similar results have also been seen when using growth hormone (71), sex steroid ablation (72), ghrelin (73), and IL-22 (74). However, although such treatment have been effective in directly enhancing thymic activity in the aged, in some instances, this may also be due, in part, by promoting hematopoiesis in the bone marrow (71, 75).

In addition to the age-related changes observed in TEC, there is an accumulation of adipose tissue particularly in the human thymus and there is increasing evidence indicating that thymic adiposity may inhibit thymic function (57). In mice, Yang and colleagues demonstrated that inducing obesity in mice accelerated thymic involution (76). In contrast, in another study, the same group observed that caloric restriction resulted in reduced thymic adiposity and delayed thymic involution (77). Although it is unclear how increase thymic adiposity alters thymic function, it has been proposed that this is due to the cytokines produced by adipocytes (57) and while involution occurs before fat deposition, suggesting that it is not initiating thymic involution, it may however exacerbate the impact of age on thymic function.

Studies have also noted an increase in the proportion of fibroblasts in the aging thymus of several species including mice (1, 54), human (52), and fish (78); suggesting that this may be a common feature. Several tissues such as heart (79), kidney (80), and liver (81) also show increased fibrosis with age which is associated with senescence and impairment of tissue function. Reports have implicated a role for TGFβ (82) and metalloproteinases (80) in the accumulation of fibroblasts in various tissues, which may be activated in response to inflammation as a result of wounding (83). It is currently unknown whether similar events also occur in the thymus, but may exacerbate the aforementioned alterations seen with age.

## Kinetics of Age-Associated Thymic Involution

An often held view is that thymic involution is triggered during puberty. This is based on studies showing that sex steroids have a detrimental effect on thymocytes and that chemical or surgical castration in older rodents is able to restore thymic size (34, 38, 64). While sex hormones are likely to contribute to thymic regression, the role of these steroids being responsible for initiating thymic involution is now being questioned (84). Indeed, several studies using a variety of thymic indices (cellularity, epithelial space, number of recent thymic emigrants) have observed that thymic involution occurs early in life, prior to puberty and that the rate of decline is not linear, but appears to be phasic. In mice, thymic cellularity begins to decrease within the first few weeks after birth (37, 45, 53, 85) and a similar picture is evident in human (51, 52, 86), equine (87), and zebrafish (88) thymus.

After this rapid early decline, involution appears to proceed at a steady rate, with studies examining human thymus suggesting a rate of 3% of thymic tissue is lost per year until middle age, followed by a rate of 1% per year (6, 89); which perhaps may cease in later life with studies showing TREC levels being barely detectable in individuals over the age of 85 years (18, 19).

Overall, these studies strongly suggest that the kinetics of age-associated thymic involution is not uniform throughout life, but characterized by distinct phases and perhaps controlled by different mechanisms. Indeed, the onset of thymic involution occurs much earlier than most acknowledged features of aging and interestingly, microarray analysis of the aged thymic revealed limited overlap with genes normally associated with aging (7). Thus, we propose that there are at least two phases in thymic involution: the first occurring in early life which would be referred to as “growth-dependent thymic involution,” as it is associated with this period of physiological growth and development and another termed “age-dependent thymic involution” linked to the age-related changes that are occurring in various body systems (85).

## Concluding Remarks

Age-associated thymic involution represents one of the most recognizable features of the aging immune system and is believed to contribute significantly toward immunosenescence. Although the molecular triggers that instigate involution remain to be fully elucidated, both intrinsic and extrinsic factors are thought to contribute toward this process. Moreover, TEC offers a potential target for rejuvenation and requires further exploration. Given the alterations in thymic development in the aged, the evidence suggests that the RTE from the aging thymus are intrinsically defective and could further exacerbate peripheral immunosenescence. Finally, additional factors that are known to modulate thymic function such as pregnancy, infection, inflammatory status, and early life events; i.e., life history is also likely to have an impact on the rate of thymic involution (9, 90).

## Conflict of Interest Statement

The author declares that the research was conducted in the absence of any commercial or financial relationships that could be construed as a potential conflict of interest.
